# Physicochemical properties of oleaster extract and the role of oleaster antioxidants on oxidative induced DNA damage

**DOI:** 10.1002/fsn3.4443

**Published:** 2024-09-03

**Authors:** Saliha Şahin, Önder Aybastıer

**Affiliations:** ^1^ Chemistry Department, Science and Arts Faculty Bursa Uludağ University Bursa Türkiye

**Keywords:** DNA oxidation, *Elaeagnus angustifolia* L., HPLC‐DAD, phenolic content, polysaccharide

## Abstract

Oleaster (*Elaeagnus angustifolia* L.) is a plant with high medicinal value and economic and nutritional importance, which has been used in traditional medicine for a long time. Oleaster contains phenolic compounds that have the ability to prevent a wide variety of diseases. In this study antioxidant capacity, total phenolic content, and total carbohydrate content were found as 108.70 ± 0.20 μg GAE/g of oleaster extract, 28.80 ± 0.01 μg TE/g of oleaster extract, and 15.40 ± 0.01 mg D‐glucose/g of oleaster extract, respectively. The oleaster extract was analyzed using the HPLC‐DAD system. The results showed rutin, caffeic acid, protocatechuic acid, and ferulic acid. The protective abilities of rutin, caffeic acid, protocatechuic acid, ferulic acid, and oleaster extract were tested against the oxidation of DNA. The mix of phenolic compounds (inhibited about 93.29% of the damage) and oleaster extract (inhibited about 94.14% of the damage) showed better protect DNA against oxidation than phenolic compounds. The results obtained from this study are guiding for new applications involving the physicochemical properties of oleaster extract with high antioxidant properties for food applications.

## Graphical abstract

Oleaster has been used as an herbal medicine due to its antioxidant, anti‐inflammatory, antimutagenic, antitumor, and anti‐arthritic properties. To investigate the physicochemical properties of oleaster and the role of oleaster antioxidants on oxidative‐induced DNA damage, oleaster extract was prepared. The results showed that oleaster extract has the ability to protect DNA against oxidation and it can be used in food application as an antioxidant agent.

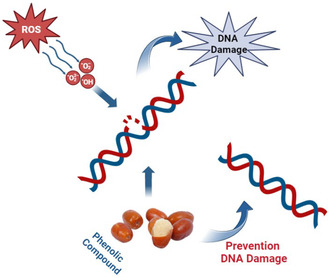

## 1 INTRODUCTION

Unstable free radicals occur in our bodies due to various internal and external effects throughout our lives. Since they are reactive, they attack cellular structures such as lipids, proteins, and DNA and cause oxidative damage to structures. DNA damage, aging, and cancer cause mutations to occur reactions in cells. The natural defense mechanisms in the body work to neutralize free radicals. However, these defense mechanisms must catch up with the increased free radicals with external factors. Therefore, consuming components with antioxidant properties is important to fight free radicals (Aybastıer et al., 2018; Jaruga et al., 2008).

The importance of natural products with many healing benefits is increasing in traditional medicine. Polysaccharides, which are natural compounds, are believed to be significant compounds with antitumor, antioxidant, hepatoprotective, antiviral, radio‐protective, and immunostimulatory activities (Jin et al., 2013; Li et al., 2013; Li & Peng, 2013). Recent studies emphasized that polysaccharides are responsible for scavenging free radicals, inhibition of viral replication, the human immune system, and lipid peroxidation (Bai et al., 2022). Flavonoids, the other important main compounds in natural products, prevent free radicals that can cause many diseases with oxidative damage in the human body (Alsawaf et al., 2022; Kolac et al., 2017). These natural antioxidants have free radical scavenging properties and reactive oxygen species interaction; inflammation related to angiogenesis, such as cancer or heart disease, causes positive effects in diseases. Flavonoids have different structural properties and various biological activities due to their different structures. Epidemiological studies have shown that increased consumption of phenolic antioxidants reduces the risk of certain types of cancer, and cardiovascular disease showed a relationship (Pinto et al., 2010; Suen et al., 2016). Also, estrogenic and anti‐estrogenic effects of flavonoids and menopausal symptoms may prevent or alleviate postmenopausal osteoporosis breast cancer. Flavonoids also inhibit proinflammatory cytokine production and receptors (Leyva‐López et al., 2016).

The most important sources of antioxidant compounds for human nutrition are health‐promoting fruits and vegetables (Alsawaf et al., 2022). These foods contain antioxidant properties due to the presence of vitamins C‐E, carotenoids, polysaccharides, and flavonoids. The consumption of high amounts of fruits and vegetables, which contain antioxidants and phytochemicals, has been linked to reduce diseases in human populations (Leyane et al., 2022). Prevent oxidative damage by neutralizing reactive oxygen species or using antioxidant compounds, which delay has been of great interest in recent years. For this purpose, natural products containing many antioxidant compounds and medicinal plants are offered for sale. The use of herbal products and plant‐derived active ingredients in the prevention of various diseases is increasing unconsciously because they are thought to be natural. Many of the antioxidant compounds found in plants have yet to be fully understood, and some active substances exhibit both oxidant and antioxidant properties. High concentrations of in vitro bacterial systems and cell cultures of some herbal products can increase oxidative damage, can be mutagenic, and can cause DNA damage, but they can be as antioxidants in combination with a synergetic effect (Aybastıer et al., 2018).

Functional and nutritional aspects of food components to improve human health are an important research topic. *Elaeagnaceae* family (*Elaeagnus angustifolia* L.) has been an herbal medicine for many years due to its medicinal properties. Due to its rich content, studies show oleaster has antioxidant, anti‐inflammatory, antimutagenic, antitussive, antitumor, and anti‐arthritic properties. It has been found to have therapeutic effects such as antimicrobial and hepatoprotective (Incilay, 2014; Ishaq et al., 2015; Khan et al., 2016; Saleh et al., 2018). As a widely grown and used plant worldwide, it is important to research oleaster in terms of its components and its effects on health. Although there are many studies on oleaster in the literature, there is no study on preventing oxidation of DNA bases.

In this study, the antioxidant abilities of oleaster extract were investigated. Total phenolic content by the Folin–Ciocalteu method, total carbohydrate content by the phenol‐sulfuric acid method, and antioxidant capacity by the ABTS method were determined. The physicochemical properties of the lyophilized oleaster extract (solubility, viscosity, and swelling capacity) were determined, and FT‐IR and SEM analyses were performed. The amounts of phenolic compounds in oleaster extract were determined by HPLC‐DAD. The protective powers of caffeic acid, ferulic acid, protocatechuic acid, rutin, and lyophilized oleaster extract against oxidatively induced DNA damage were determined by GC–MS/MS by analyzing DNA base damage products. This study is believed to provide information about oleaster's physicochemical and functional properties for their food applications and its ability for protecting biomolecules, such as DNA, against oxidation.

## 2 MATERIALS AND METHODS

### 2.1 Materials

Ethanol, 2‐propanol, potassium persulfate, iron (II) sulfate‐7‐hydrate, TMCS, and formic acid were purchased from Merck (Darmstadt, Germany). Acetonitrile, ABTS, sodium hydroxide, Folin–Ciocalteu reagent, copper(II) sulfate pentahydrate, potassium sodium tartrate, sodium carbonate, caffeic acid, ferulic acid, gallic acid, trolox, rutin, protocatechuic acid in HPLC grade. Water was purified with Purelab Option‐Q from Elga Laboratory (UK). 56DHU (5,6‐dihydrouracil) and pyridine were purchased from Sigma‐Aldrich (St. Louis, MO., USA). BSTFA and H_2_O_2_ were purchased from Fluka (Switzerland). Lyophilized DNA powder from calf thymus, D‐glucose, and phenol were purchased from Sigma (Germany). Alx (alloxane) was obtained from Titan Biotech (India). 5FU (5‐formyluracil) was obtained from IS Chemical Technology in China. 5HMU (5‐(hydroxymethyl)uracil) and were obtained from Aldrich Chemical (USA). 5HC (5‐hydroxycytosine), 5H5MH (5‐hydroxy‐5‐methylhydantoin), and 5HH (5‐hydroxyhydantoin) were obtained from Toronto Research Chemicals (Canada). 5HU (5‐hydroxyuracil), TG (thymine glycol), 5HMC (5‐(hydroxymethyl)cytosine), and 8HA (8‐hydroxyadenine) were purchased from the National Institute of Standards and Technology (USA). Fapy‐ade (4,6‐Diamino‐5‐(formylamino)pyrimidine) was taken from Santa Cruz Biotechnology (Texas, USA). Oleaster (Elaeagnus angustifolia L.) was purchased from local market from Bursa in Turkey.

### 2.2 Extraction method

Twenty gram of oleaster without peel and seed was extracted with 100 mL of ethanol‐water (1:3, v/v) solvent mixture for 4 h at 25°C. After filtering, the residue was ultrasonic‐assisted extraction (37 Hz) with 100 mL of distilled water at 6 h at 60°C. At the end of each hour, the extract was collected from the extract media and stored at 4°C for analysis. These extracts were used for other analyses.

### 2.3 Total carbohydrate analysis

Total carbohydrate analysis was performed on the extracts collected every hour. The analysis was carried out using the phenol‐sulfuric acid method. One milliliter of extract sample and 3 mL of concentrated sulfuric acid were added and vortexed for 30 s. Then, 1 mL of 5% (w/v) phenol was added. After vortexing, the samples were incubated in a water bath at 90°C for 5 min. If the phenol solution is added to the carbohydrates in the presence of sulfuric acid, a colored complex is formed. The absorbance of this complex was measured with a UV–VIS spectrophotometer at 490 nm (Sharifian‐Nejad & Shekarchizadeh, 2019). For this purpose, standard solutions with different concentrations from 1% D‐glucose were prepared, and a calibration graph was drawn as a result of the measurements.

### 2.4 Total phenolic content

The total phenolic content of extracts was determined by the Folin–Ciocalteu method (Şahin et al., 2015). Lowry A solution with 2% Na_2_CO_3_ (%, w/v) in 0.1 M NaOH and 0.5% CuSO_4_ (%, w/v) in 1% NaKC_4_H_4_O_6_ (%, w/v) Lowry B solution was prepared. Lowry A and Lowry B are mixed in a 50:1 (v/v) ratio, and Lowry C solution was prepared. 0.1 mL sample/standard and 1.9 mL distilled water were added to the analysis tubes, followed by 2.5 mL of Lowry C solution and 0.25 mL of Folin–Ciocalteu reagent (diluted 1:3 ratio with water). After standing in the dark for 30 min, the absorbance of samples and standards at 750 nm was measured. A calibration graph of gallic acid versus absorbance was drawn. The absorbance of the samples was replaced in the graphic equation, and the total phenolic content was determined in terms of μg gallic acid equivalent (GAE)/g of oleaster extract.

### 2.5 Antioxidant capacity

ABTS method was used for the determination antioxidant capacity of samples (Şahin et al., 2015). 20.0 mM ABTS and 2.45 mM K_2_S_2_O_8_ were dissolved in water. It was kept in the dark for 24 h, and then the ABTS solution was diluted 1:10 with water. 0.1 mL of sample/standard, 3.9 mL of ethanol, and 1 mL of diluted ABTS solution were added to the tubes. Then, absorbances were measured at 734 nm (*A*_sample_). The %inhibition value of each sample was calculated based on the absorbance of the antioxidant‐free sample (*A*_blank_).

$$\%\text{Inhibition}=\left(A_{\text{blank}}-A_{\text{sample}}\right)\times 100/A_{\text{blank}}$$

The antioxidant capacity amounts for the samples were calculated as “μg trolox equivalent (TE)/g of oleaster extract” using the determined calibration equation.

### 2.6 Viscosity and swelling capacity

A weight of 0.5 g was taken from the lyophilized residue. Then, 10.0 mL of distilled water was added to the measuring cylinder, and the volume (*V*_1_) was recorded. It was stored at room temperature, and the swollen mass's sediment volume (*V*_2_) was noted after 24 h. The swelling capacity was calculated by determining the ratio of the swollen volume to the initial bulk volume using the formula (Lourith & Kanlayavattanakul, 2017).

$$\%\text{Swelling capacity}=V_{2}/V_{1}\times 100$$

The lyophilized sample (0.3 g) was saturated in water (7.5 mL) for 24 h, adjusted to 10 mL with water, and mixed thoroughly at 150 rpm under room temperature for 2 h. The viscosity of the resulting solution was determined by the Ostwald viscometer (Lourith & Kanlayavattanakul, 2017).

### 2.7 HPLC analysis

For the quantitative determination of phenolic substances found in oleaster extract, HPLC‐DAD was used. For analysis with HPLC‐DAD, 10 μL injection volume and 0.5 mL/min flow rate were studied. A gradient mobile phase program consisting of 1% aqueous formic acid and acetonitrile was implemented during the analysis. Operating conditions for HPLC‐DAD were given in Table 1. After the solutions of the phenolic standards were prepared, they were analyzed by HPLC‐DAD. Linear equations were calculated using the least squares method by drawing graphs of the peak area against the concentration. The peak area values of the samples were replaced in the equation, and the phenolic substances in the oleaster were calculated in terms of μg/g of oleaster extract.

**TABLE 1:** HPLC‐DAD gradient program.

Time (min)	Mobile phase (%)	
	1% aqueous formic acid	Acetonitrile
0	90	10
10	87	13
20	58.5	41.5
25	30	70
35	90	10
36	90	10

### 2.8 Investigation of the prevention of DNA damage

The required oxidative stress environment was created using the Fenton reaction. H_2_O_2_, FeSO_4_, caffeic acid, rutin, protocatechuic acid, and ferulic acid stock solutions were prepared. Eight samples are required to examine the antioxidant effects against DNA oxidation. The volume of each sample is 2500 μL and consists of 100 μg DNA in addition to the other ingredients described as: (1) 100 μg DNA only, (2) Fenton (100 μg DNA, 200 μM H_2_O_2_, and 100 μM Fe^2+^), (3) Fenton and 4.95 μM caffeic acid, (4) Fenton and 2.51 μM ferulic acid, (5) Fenton and 7.42 μM protocatechuic acid, (6) Fenton and 10.67 μM rutin, (7) Fenton and standard mix (equivalent to 4.95 μM caffeic acid, 2.51 μM ferulic acid, 7.42 μM protocatechuic acid, and 10.67 μM rutin), (8) Fenton and extract (includes 4.95 μM caffeic acid, 2.51 μM ferulic acid, 7.42 μM protocatechuic acid, and 10.67 μM rutin). Each preparation starts with 100 μg of DNA being placed in a glass tube, ultra‐pure water, antioxidant (or extract), H_2_O_2_, and FeSO_4_ (Fe^2+^) were added sequentially, and all used solutions were prepared by calculating the desired concentrations from the previously prepared stock solutions. After thoroughly mixing the contents of the samples, they were well capped and kept in the incubator at 37°C for 20 min. The samples were then frozen at −20°C and then lyophilized in a lyophilizer with a cooling system at −86°C under 0.1 mbar vacuum for 24 h. 1 mL of 60% (v/v) aqueous formic acid was added to each sample. The samples were heated at 130°C in the oven for 30 min to hydrolyze the samples. DNA base damage products were released through hydrolysis as free bases that can be detected (Aybastıer & Demir, 2021; Jaruga et al., 2008). The samples were then frozen and lyophilized under the same conditions as before. Lyophilized samples were derivatized using 1% TMCS in BSTFA. Derivatization was completed in a nitrogen atmosphere at 120°C for 40 min. The analysis was performed using a Gas Chromatography (Trace 1300) triple quadrupole mass spectrometer (TSQ 8000 Evo) from Thermo Scientific (USA) according to the literature (Aybastıer & Demir, 2021; Şahin et al., 2020).

### 2.9 FT‐IR and SEM analysis

The lyophilized oleaster extract was characterized using ATR on a Perkin Elmer (Spectrum 100, USA) spectrometer at 500–4000 cm^−1^. The surface morphology of lyophilized oleaster extract was studied using a scanning electron microscope (SEM, Carl Zeiss Evo 40, Germany) at 10–20 kV. The samples were mounted on aluminum stubs and sputter‐coated with gold.

## 3 RESULTS AND DISCUSSION

### 3.1 Physiochemical and antioxidant properties of oleaster

**FIGURE 1:**
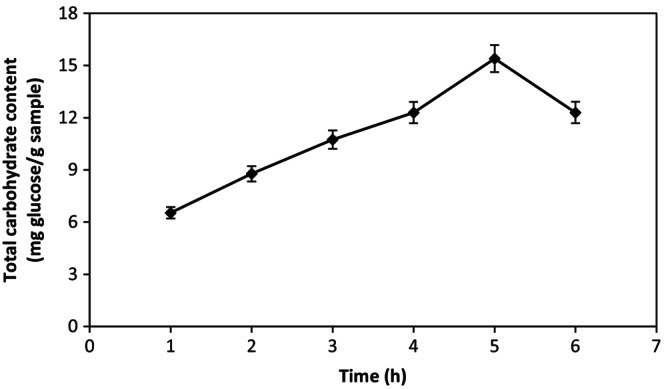
The changing of total carbohydrate content of oleaster extract with extraction time.

The effect of different extraction times on total carbohydrate content (TCC) was investigated in this study. Comparative extraction times (1–6 h) indicated that 5 h afforded the higher TCC than extracts obtained after 6, 4, 3, 2, and 1 h for oleaster extract (Figure 1). The extraction time was chosen 5 h for extraction of polysaccharide from oleaster extract because of the highest TCC for 5 h. TCC of the aqueous extract was higher than in literature (Lourith & Kanlayavattanakul, 2017) because oleaster extract contains monosaccharides and polysaccharides. We investigated the oleaster extract's physicochemical and antioxidant properties (Table 2). Significant variations in total phenolic content (TPC) and antioxidant capacities (AC) of oleaster extract were observed. TPC extracted by different extraction times was 81.11 and 108.70 μg GAE/g of oleaster extract for 1 and 5 h, respectively. TPC of the aqueous extract for 5 h was relatively high, possibly because oleaster extract contains higher flavonoid glycosides than TCC. AC extracted by different extraction times was 19.60 and 28.80 μg TE/g of oleaster extract for 1 and 5 h, respectively. The aqueous oleaster extract for 5 h showed the highest ability to scavenge ABTS radical cation. The best extraction time was 5 h for the highest TCC, TPC, and AC for oleaster. The validation of swelling capacity and viscosity parameters is important when extracts can be used in cosmetic applications. As the swelling capacity of the hydrogels increases, the hydrophilic property of the hydrogel network increases (Archana et al., 2013). As can be seen, the polysaccharide extract in the oleaster extract for 5 h hydrated and formed a viscous solution, increasing water swelling (2.01 ± 0.01%). The viscosity of the oleaster extract varies with the swelling capacity. The viscosity value of the extract for 5 h was found to be 1067 ± 16 cps in this study. Accordingly, oleaster extract can be used in different areas as a thickening and suspending agent.

**TABLE 2:** Physicochemical and spectroscopic properties of oleaster extract.

Property	Value
Viscosity (cps)	1067 ± 16
Swelling capacity in water (%)	2.01 ± 0.01
Total phenolic content after 5 h extraction (μg GAE/g sample)	108.70 ± 0.20
Total phenolic content after 1 h extraction (μg GAE/g sample)	81.11 ± 1.41
Antioxidant capacity after 5 h extraction (μg TE/g sample)	28.80 ± 0.01
Antioxidant capacity after 1 h extraction (μg TE/g sample)	19.60 ± 0.07

### 3.2 FT‐IR and SEM analysis of oleaster extract

**FIGURE 2:**
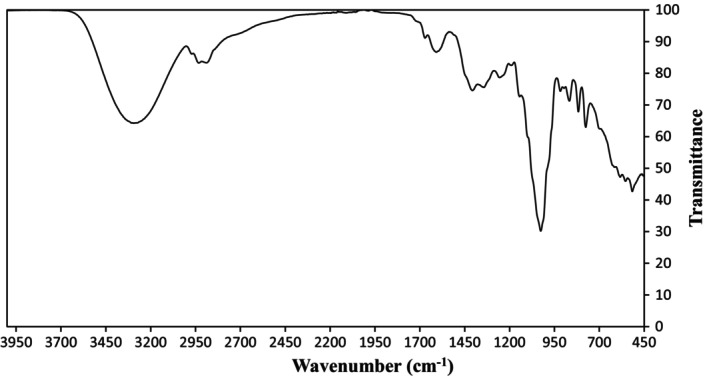
FT‐IR spectrum of oleaster extract.

**FIGURE 3:**
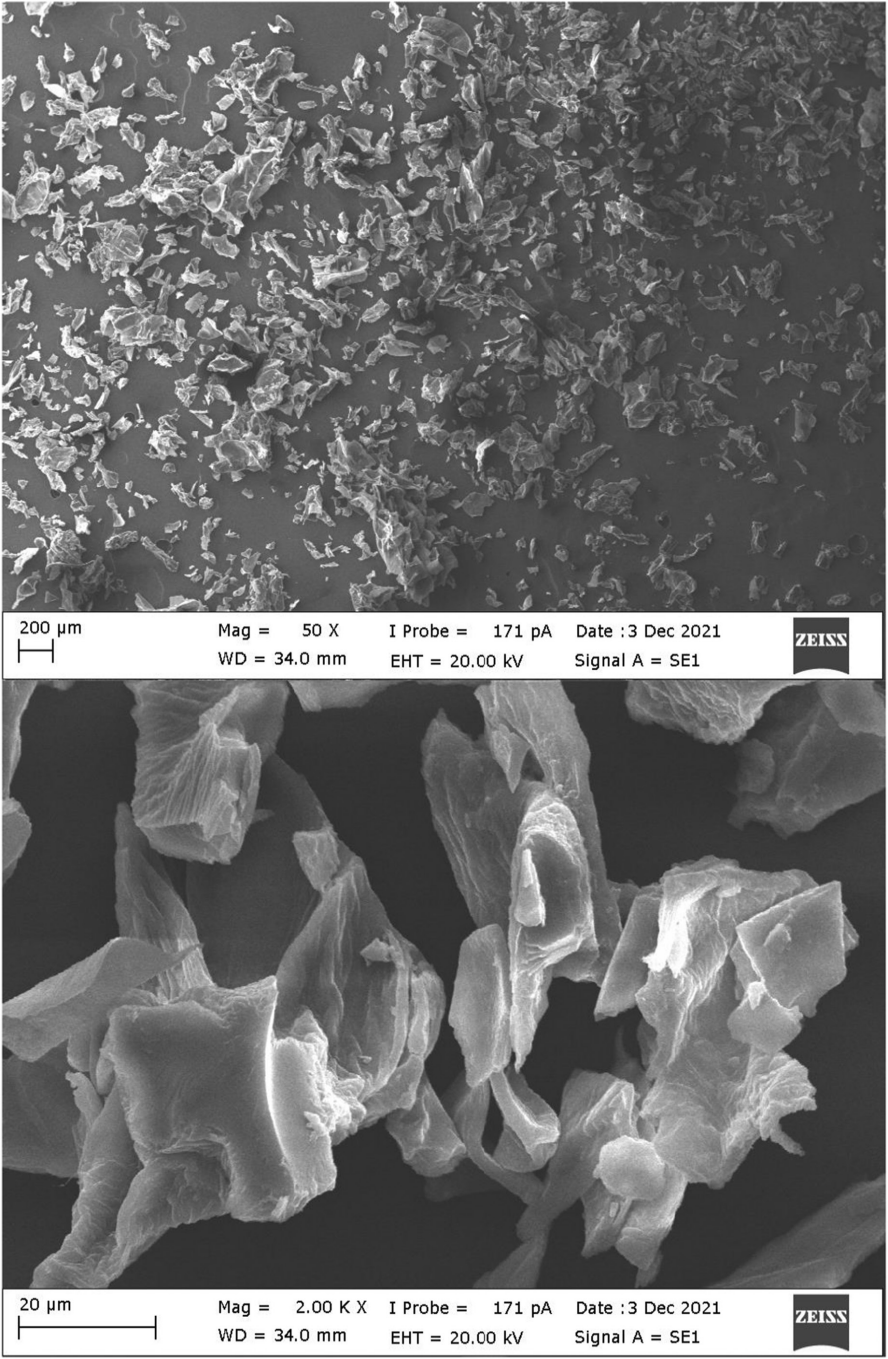
SEM surface micrographs of oleaster extract.

Structural analysis of oleaster extract for 5 h was achieved using FTIR and SEM. The FTIR spectrum of lyophilized oleaster extract is seen in Figure 2. The characteristic absorption bands are present at 1274 cm^−1^ (C‐O), 1366 cm^−1^ (O‐H), 1423 cm^−1^ (COO‐), 1627 cm^−1^ (‐COOR), 2948 cm^−1^ (C‐H), and 3333 cm^−1^ (O‐H). Due to the large number of hydrogen bonds related to hydroxyls of different secondary metabolites, the viscosity of the oleaster extract has increased, and accordingly (Archana et al., 2013), its potential for use in related industrial applications has increased. SEM can be used to examine the surface structure. According to SEM images seen in Figure 3, clusters with sharp angles similar to those in the literature were observed on the lyophilized oleaster extract, which is an amorphous solid. The increase in surface area may increase the solubility of the product due to its irregular size and form. These properties can be seen in lyophilized extracts.

### 3.3 HPLC analysis of oleaster extract

In the quantitative analysis of phenolic substances in the oleaster extract by HPLC‐DAD, it was determined that oleaster extract for 5 h contained caffeic acid, protocatechuic acid, ferulic acid, and rutin. Rutin was the most abundant, with 32.58 μg/g of oleaster extract. Free benzoic acid derivatives can be found in fruits. Cinnamic acid types such as coumaric acid and caffeic acid are commonly found in pears, grapes, and apples (Spanos & Wrolstad, 1992). However, basic phenolics are characteristic compounds of fruit samples. Protocatechuic acid, with a level of 6.08 μg/g of oleaster extract, was found to be the second most abundant acid in the oleaster extract. Caffeic (4.46 μg/g of oleaster extract) and ferulic acid (2.44 μg/g of oleaster extract) were also found in comparatively high amounts.

### 3.4 The ability to prevent DNA oxidation of oleaster extract

Antioxidant‐type compounds are expected to protect the content and nutritional quality of foods. However, antioxidants play a role in preventing diseases that cause much tissue damage. Antioxidants prevent cancer, cardiovascular disease, and some oxidative stress diseases (Unuofin & Lebelo, 2020). The oleaster extract contained significant antioxidant properties and phytochemical compounds. The ability to prevent DNA oxidation of oleaster extract was investigated by tracing 12 DNA base damage products by GC–MS/MS analysis with induced DNA oxidation. Oxidatively induced DNA base damage product values (ng/mg DNA) were found statistically significant (*p* < .01) in Table 3.

**TABLE 3:** Oxidatively induced DNA base damage products (ng/mg DNA).

Compounds	DNA	Fenton	CA	FA	PCA	RU	Mix	Ext
56DHU	<LOD	1.89 ± 0.15^a^	1.67 ± 0.12^c^	0.36 ± 0.05^b^	0.87 ± 0.06^c^	0.39 ± 0.0^c^	0.24 ± 0.02^b^	0.18 ± 0.02^c^
5H5MH	1.76 ± 0.11^g^	41.18 ± 2.74^j^	5.78 ± 0.66^k^	18.12 ± 1.01^k^	5.34 ± 0.68^j^	8.87 ± 0.56^L^	7.64 ± 0.51^L^	3.94 ± 0.36^j^
5HH	2.16 ± 0.14^h^	40.48 ± 3.55^h^	2.60 ± 0.15^e^	3.96 ± 0.32^h^	2.38 ± 0.21^g^	3.63 ± 0.21^i^	5.16 ± 0.48^k^	2.50 ± 0.19^h^
5FU	0.24 ± 0.02^c^	42.55 ± 3.83^k^	3.44 ± 0.21^g^	2.24 ± 0.18^g^	1.68 ± 0.09^e^	2.10 ± 0.12^g^	0.70 ± 0.03^d^	2.42 ± 0.11^g^
5HU	0.74 ± 0.01^d^	163.74 ± 8.48^L^	5.09 ± 0.28^j^	12.66 ± 0.98^j^	7.90 ± 0.62^L^	2.40 ± 0.06^h^	3.53 ± 0.18^j^	4.95 ± 0.29^k^
5HMU	1.29 ± 0.05^f^	35.83 ± 2.16^g^	2.84 ± 0.25^f^	9.61 ± 0.56_i_	5.48 ± 0.64^k^	1.74 ± 0.15^e^	2.56 ± 0.14^i^	0.42 ± 0.03^e^
Alx	0.87 ± 0.04^e^	10.25 ± 0.88^d^	3.69 ± 0.19^h^	2.05 ± 0.21^e^	3.72 ± 0.24^h^	3.69 ± 0.20^j^	2.00 ± 0.05^g^	0.12 ± 0.01^b^
5HC	<LOD	16.12 ± 1.02^f^	4.72 ± 0.35^i^	0.72 ± 0.08^c^	5.18 ± 0.48^i^	4.64 ± 0.32^k^	0.12 ± 0.01^a^	0.35 ± 0.02^d^
TG	0.22 ± 0.02^b^	3.16 ± 0.21^b^	0.16 ± 0.02^a^	<LOD	0.17 ± 0.01^b^	0.24 ± 0.01^a^	0.37 ± 0.03^c^	0.07 ± 0.01^a^
5HMC	<LOD	16.05 ± 0.95^e^	<LOD	0.10 ± 0.01^a^	0.12 ± 0.01^a^	0.32 ± 0.02^b^	1.84 ± 0.08^f^	2.80 ± 0.22^i^
FapyAde	0.15 ± 0.01^a^	8.42 ± 0.52^c^	0.74 ± 0.03^b^	2.11 ± 0.15^f^	0.97 ± 0.02^d^	1.42 ± 0.09^d^	2.37 ± 0.11^h^	5.56 ± 0.41^L^
8HA	<LOD	40.55 ± 2.11^i^	2.04 ± 0.11^d^	1.96 ± 0.21^d^	1.78 ± 0.16^f^	2.02 ± 0.17^f^	1.64 ± 0.15^e^	1.30 ± 0.10^f^
Total	7.43 ± 0.19	420.22 ± 10.90	32.78 ± 0.91	53.88 ± 1.60	35.59 ± 1.28	31.46 ± 0.76	28.18 ± 0.77	24.62 ± 0.70

*Note*: Two replicates±standard deviation. Fenton: 200 μM H_2_O_2_ and 100 μM Fe^2+^, CA: caffeic acid (4.95 μM); FA: ferulic acid (2.51 μM); PCA: protocatechuic acid (7.42 μM); RU: rutin (10.67 μM); Mix: 4.95 μM caffeic acid, 2.51 μM ferulic acid, 7.42 μM protocatechuic acid and 10.67 μM rutin. Different lowercase letters (a‐l) indicate significant differences between results (*p* < .01).

Abbreviations: 56DHU: 5;6‐dihydrouracil; 5FU: 5‐formyluracil; 5H5MH: 5‐hydroxy‐5‐methylhydantoin; 5HC: 5‐hydroxycytosine; 5HH: 5‐hydroxy hydantoin; 5HMC: 5‐(hydroxymethyl)cytosine; 5HMU: 5‐(hydroxymethyl)uracil; 5HU: 5‐hydroxyuracil; 8HA: 8‐hydroxyadenine; Alx: alloxane; FapyAde: 4,6‐diamino‐5‐(formylamino)pyrimidine; LOD: limit of detection; TG: thymine glycol.

**FIGURE 4:**
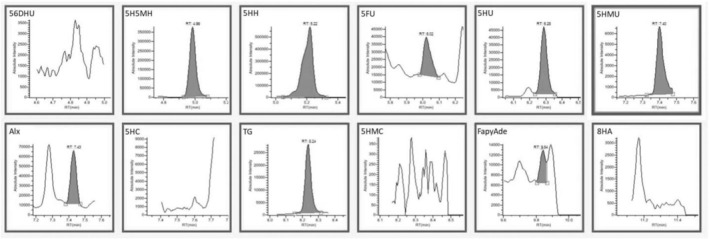
GC–MS/MS chromatograms of DNA damage products without induced oxidation. 56DHU, 5;6‐dihydrouracil; 5FU, 5‐formyluracil; 5H5MH, 5‐hydroxy‐5‐methylhydantoin; 5HC, 5‐hydroxycytosine; 5HH, 5‐hydroxy hydantoin; 5HMC, 5‐(hydroxymethyl)cytosine; 5HMU, 5‐(hydroxymethyl)uracil; 5HU, 5‐hydroxyuracil; 8HA, 8‐hydroxyadenine; Alx, alloxane; FapyAde, 4,6‐diamino‐5‐(formylamino)pyrimidine; TG, thymine glycol.

**FIGURE 5:**
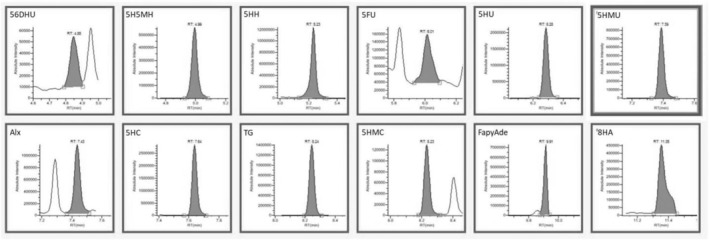
GC–MS/MS chromatograms of DNA damage products with induced oxidation (Fenton reaction). 56DHU, 5;6‐dihydrouracil; 5FU, 5‐formyluracil; 5H5MH, 5‐hydroxy‐5‐methylhydantoin; 5HC, 5‐hydroxycytosine; 5HH, 5‐hydroxy hydantoin; 5HMC, 5‐(hydroxymethyl)cytosine; 5HMU, 5‐(hydroxymethyl)uracil; 5HU, 5‐hydroxyuracil; 8HA, 8‐hydroxyadenine; Alx, alloxane; FapyAde, 4,6‐diamino‐5‐(formylamino)pyrimidine; TG, thymine glycol.

**FIGURE 6:**
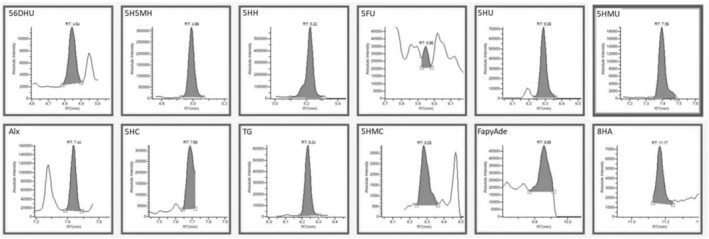
GC–MS/MS chromatograms of DNA damage products obtained in mixed 4.95 μM caffeic acid, 2.51 μM ferulic acid, 7.42 μM protocatechuic acid, and 10.67 μM rutin sample with induced oxidation. 56DHU: 5;6‐dihydrouracil; 5FU: 5‐formyluracil; 5H5MH: 5‐hydroxy‐5‐methylhydantoin; 5HC: 5‐hydroxycytosine; 5HH: 5‐hydroxy hydantoin; 5HMC: 5‐(hydroxymethyl)cytosine; 5HMU: 5‐(hydroxymethyl)uracil; 5HU: 5‐hydroxyuracil; 8HA: 8‐hydroxyadenine; Alx: alloxane; FapyAde: 4,6‐diamino‐5‐(formylamino)pyrimidine; TG: thymine glycol.

**FIGURE 7:**
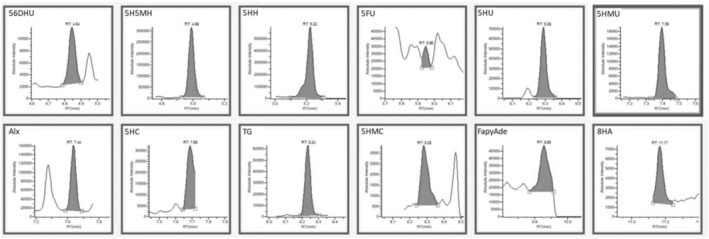
GC–MS/MS chromatograms of DNA damage products obtained in mixed oleaster extract with induced oxidation. 56DHU, 5;6‐dihydrouracil; 5FU, 5‐formyluracil; 5H5MH, 5‐hydroxy‐5‐methylhydantoin; 5HC, 5‐hydroxycytosine; 5HH, 5‐hydroxy hydantoin; 5HMC, 5‐(hydroxymethyl)cytosine; 5HMU, 5‐(hydroxymethyl)uracil; 5HU, 5‐hydroxyuracil; 8HA, 8‐hydroxyadenine; Alx, Alloxane; FapyAde, 4,6‐diamino‐5‐(formylamino)pyrimidine; TG, Thymine glycol.

According to Table 3, the lowest concentration of DNA base damage products was obtained in the control DNA when Fenton oxidation was not applied. As a result of GC–MS/MS analysis shown in Figure 4, 7.43 ng/mg of DNA base damage product was determined in the control DNA sample. This value increased approximately 60‐fold when the DNA sample was subjected to Fenton oxidation (100 μg DNA, 200 μM H_2_O_2_, and 100 μM Fe^2+^) (Figure 5). As antioxidant substances and extract were added to DNA, the damaged products decreased. Better data on DNA oxidative damage can be obtained by comparing the total amounts of DNA base damage products. This study investigated the ability of caffeic acid, ferulic acid, protocatechuic acid, and rutin to protect DNA bases from oxidation. Compared to the Fenton control sample, 4.95 μM caffeic acid concentration reduced approximately 92% of DNA damage, 2.51 μM ferulic acid reduced approximately 87%, and 7.42 μM protocatechuic acid concentration was approximately 91% of DNA damage. 10.67 μM can prevent approximately 92% of routine damage. When a mixture of these antioxidants and an equivalent extract was used, DNA base damage production in DNA samples subjected to oxidation by the Fenton reaction decreased to 28.18–24.62 ng/mg DNA levels, respectively. DNA base damage products were significantly reduced (Figures 6 and 7). The ability of antioxidants to protect DNA bases from oxidation varies depending on the antioxidant type and concentration. The total DNA base damages products; when antioxidants are used as a mixture, they are lower than when used separately. In this case, it is thought that it may cause antagonism in the antioxidant system in preventing damage production. In addition, oleaster extract equivalent to 4.95 μM caffeic acid, 2.51 μM ferulic acid, 7.42 μM protocatechuic acid, and 10.67 μM rutin caused more than 95% inhibition of DNA base damage. In this situation, it was thought that caffeic acid, ferulic acid, protocatechuic acid, and different antioxidant substances determined in oleaster extract and rutin contributed to the high DNA base damage prevention percentage of oleaster extract.

## 4 CONCLUSIONS

The evaluation of the physicochemical properties of oleaster extract and its ability to inhibit the oxidatively induced damage of DNA was studied. It can be concluded that the oleaster extract contained significant antioxidant capacity and phytochemical compounds (caffeic acid, ferulic acid, protocatechuic acid, and rutin). Oleaster extract also showed antioxidant activity against Fenton‐induced DNA oxidation. The damage inhibition by oleaster was better than that of the mixed caffeic acid, ferulic acid, protocatechuic acid, and rutin. Competitive antagonism is thought to be the mechanism by which the effect of mixed antioxidants is decreased. As a result, oleaster extract can protect biomolecules, particularly DNA, against oxidation. Also, that extract can be used as a suspended antioxidant agent in food applications.

## AUTHOR CONTRIBUTIONS

**Saliha Şahin:** Conceptualization (equal); data curation (equal); formal analysis (equal); funding acquisition (equal); investigation (equal); methodology (equal); project administration (equal); resources (equal); software (equal); supervision (equal); validation (equal); visualization (equal); writing – original draft (equal). **Önder Aybastıer:** Conceptualization (equal); data curation (equal); formal analysis (equal); funding acquisition (equal); investigation (equal); methodology (equal); project administration (equal); resources (equal); software (equal); supervision (equal); validation (equal); visualization (equal); writing – original draft (equal).

## Data Availability

Not available.
